# 3-Eth­oxy­methyl-1,4-dihydro­quinolin-4-one

**DOI:** 10.1107/S1600536812028383

**Published:** 2012-06-27

**Authors:** Wafaa A. Zaghary, Reem I. Al-Wabli, Seik Weng Ng

**Affiliations:** a Department of Pharmaceutical Chemistry, College of Pharmacy, King Saud University, Riyadh 11451, Saudi Arabia; b Department of Chemistry, University of Malaya, 50603 Kuala Lumpur, Malaysia; c Chemistry Department, Faculty of Science, King Abdulaziz University, PO Box 80203 Jeddah, Saudi Arabia

## Abstract

In the title mol­ecule, C_12_H_13_NO_2_, the dihydro­quinolinone fused-ring system is nearly planar [maximum deviation = 0.012 (3) Å], and the mean plane passing through the extended eth­oxy­methyl substituent is aligned at 86.9 (2)° with respect to the fused-ring system. In the crystal, adjacent mol­ecules are linked by an N—H⋯O_carbon­yl_ hydrogen bond to generate a chain running along the *b*-axis direction.

## Related literature

For the crystal structure of 1,4-dihydro­quinolin-4-one, see: Nasiri *et al.* (2006 ▶).
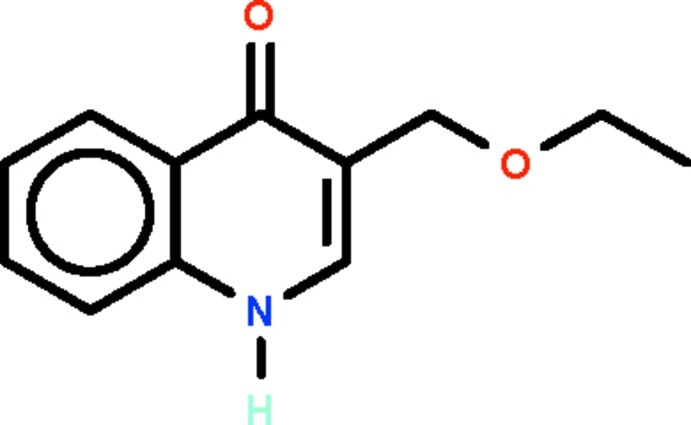


## Experimental

### 

#### Crystal data

C_12_H_13_NO_2_
*M* *_r_* = 203.23
Orthorhombic, 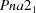

*a* = 18.179 (3) Å
*b* = 12.4052 (16) Å
*c* = 4.4529 (5) Å
*V* = 1004.2 (2) Å^3^
*Z* = 4
Mo *K*α radiation
μ = 0.09 mm^−1^
*T* = 100 K
0.35 × 0.05 × 0.03 mm

#### Data collection

Agilent SuperNova Dual diffractometer with an Atlas detector
Absorption correction: multi-scan (*CrysAlis PRO*; Agilent, 2012 ▶) *T*_min_ = 0.969, *T*_max_ = 0.997
3157 measured reflections
1306 independent reflections
913 reflections with *I* > 2σ(*I*)
*R*_int_ = 0.068

#### Refinement

*R*[*F* ^2^ > 2σ(*F* ^2^)] = 0.055
*wR*(*F* ^2^) = 0.116
*S* = 1.01
1306 reflections
140 parameters
1 restraint
H atoms treated by a mixture of independent and constrained refinement
Δρ_max_ = 0.24 e Å^−3^
Δρ_min_ = −0.24 e Å^−3^

### 

Data collection: *CrysAlis PRO* (Agilent, 2012 ▶); cell refinement: *CrysAlis PRO*; data reduction: *CrysAlis PRO*; program(s) used to solve structure: *SHELXS97* (Sheldrick, 2008 ▶); program(s) used to refine structure: *SHELXL97* (Sheldrick, 2008 ▶); molecular graphics: *X-SEED* (Barbour, 2001 ▶); software used to prepare material for publication: *publCIF* (Westrip, 2010 ▶).

## Supplementary Material

Crystal structure: contains datablock(s) global, I. DOI: 10.1107/S1600536812028383/xu5578sup1.cif

Structure factors: contains datablock(s) I. DOI: 10.1107/S1600536812028383/xu5578Isup2.hkl

Supplementary material file. DOI: 10.1107/S1600536812028383/xu5578Isup3.cml

Additional supplementary materials:  crystallographic information; 3D view; checkCIF report

## Figures and Tables

**Table 1:** Hydrogen-bond geometry (Å, °)

*D*—H⋯*A*	*D*—H	H⋯*A*	*D*⋯*A*	*D*—H⋯*A*
N1—H1⋯O1^i^	0.98 (4)	1.78 (4)	2.707 (3)	157 (4)

Symmetry code: (i) 
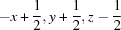
.

**Fig. 1.**
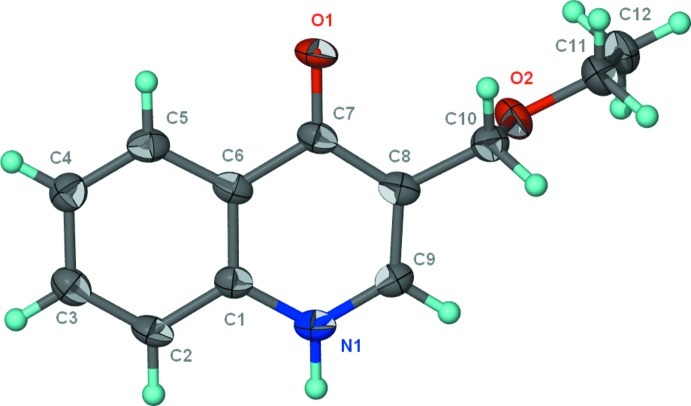
Thermal ellipsoid plot (Barbour, 2001) of C12H13NO2 at the 70% probability level; H atoms are drawn as spheres of arbitrary radius.

**Fig. 2.**
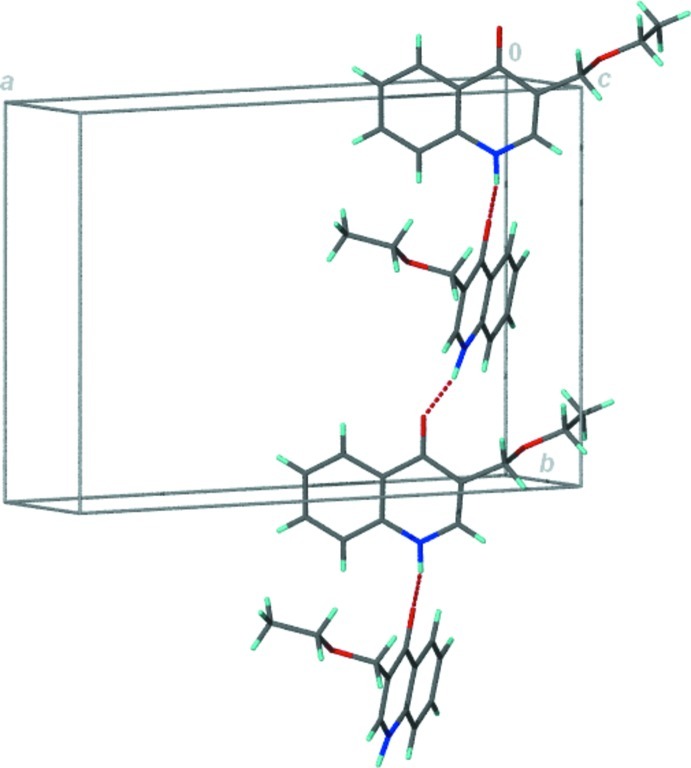
Hydrogen-bonded chain structure.

## supplementary crystallographic information

### Comment

The title compound was the unexpected product of the Mannich reaction involving 4-hydroxyquinoline, morpholine and paraformaldehyde in ethanol medium in an attempt to place the O(CH_2_CH_2_)N–CH_2_– unit at the 3-position of 4-hydroxyquinoline. The compound was to have been radiolabeled with ^99^*^m^*Tc for an imaging study. The ethyl group in the unexpected product (Scheme I) probably come from the ethanol solvent. The mean plane passing through the extended ethoxymethyl substituent is aligned at 86.9 (2) ° with respect to the mean plane passing through the dihydroquinoline fused-ring (Fig. 1). Adjacent molecules are linked by an N—H···O_carbonyl_ hydrogen bond to generate a chain running along the *b*-axis of the orthorhombic unit cell (Table 1).

### Experimental

A mixture of paraformaldehyde (0.18 g, 0.002 mole) and morpholine (0.174 g, 0.002 mole) in alcohol (15 ml) was heated for 30 min. A solution of 4-quinolinol (0.29 g, 0.002 mole) in ethanol (5 ml) was added and the mixture was heated for 24 h. The solid that formed collected and purified by coloumn chromatography. Colorless crystals were obtained upon recrystallization from toluene/ethanol (9:1).

### Refinement

Carbon-bound H atoms were placed in calculated positions [C—H 0.95 to 0.98 Å, *U*_iso_(H) 1.2 to 1.5*U*_eq_(C)] and were included in the refinement in the riding model approximation.

The amino H atom was located in a difference Fourier map, and was refined.

In the absence of heavy scatterers, 487 Friedel pairs were merged.

### Crystal data

**Table:**

C_12_H_13_NO_2_	*F*(000) = 432
*M**_r_* = 203.23	*D*_x_ = 1.344 Mg m^−^^3^
Orthorhombic, *P**n**a*2_1_	Mo *K*α radiation, λ = 0.71073 Å
Hall symbol: P 2c -2n	Cell parameters from 568 reflections
*a* = 18.179 (3) Å	θ = 2.8–27.5°
*b* = 12.4052 (16) Å	µ = 0.09 mm^−^^1^
*c* = 4.4529 (5) Å	*T* = 100 K
*V* = 1004.2 (2) Å^3^	Prism, colourless
*Z* = 4	0.35 × 0.05 × 0.03 mm

### Data collection

**Table:**

Agilent SuperNova Dual diffractometer with an Atlas detector	1306 independent reflections
Radiation source: SuperNova (Mo) X-ray Source	913 reflections with *I* > 2σ(*I*)
Mirror monochromator	*R*_int_ = 0.068
Detector resolution: 10.4041 pixels mm^-1^	θ_max_ = 27.5°, θ_min_ = 2.8°
ω scans	*h* = −23→19
Absorption correction: multi-scan (*CrysAlis PRO*; Agilent, 2012)	*k* = −9→16
*T*_min_ = 0.969, *T*_max_ = 0.997	*l* = −5→4
3157 measured reflections	

### Refinement

**Table:**

Refinement on *F*^2^	Primary atom site location: structure-invariant direct methods
Least-squares matrix: full	Secondary atom site location: difference Fourier map
*R*[*F*^2^ > 2σ(*F*^2^)] = 0.055	Hydrogen site location: inferred from neighbouring sites
*wR*(*F*^2^) = 0.116	H atoms treated by a mixture of independent and constrained refinement
*S* = 1.01	*w* = 1/[σ^2^(*F*_o_^2^) + (0.0411*P*)^2^] where *P* = (*F*_o_^2^ + 2*F*_c_^2^)/3
1306 reflections	(Δ/σ)_max_ = 0.001
140 parameters	Δρ_max_ = 0.24 e Å^−^^3^
1 restraint	Δρ_min_ = −0.24 e Å^−^^3^

### Fractional atomic coordinates and isotropic or equivalent isotropic displacement parameters (Å^2^)

**Table:**

	*x*	*y*	*z*	*U*_iso_*/*U*_eq_	
O1	0.27733 (13)	0.81501 (17)	0.7508 (6)	0.0265 (6)	
O2	0.09593 (14)	0.90044 (19)	0.8715 (5)	0.0280 (6)	
N1	0.25633 (17)	1.1355 (2)	0.5678 (7)	0.0225 (7)	
C1	0.3120 (2)	1.0792 (3)	0.4303 (7)	0.0197 (8)	
C2	0.3589 (2)	1.1317 (3)	0.2279 (8)	0.0225 (8)	
H2	0.3530	1.2064	0.1877	0.027*	
C3	0.4137 (2)	1.0741 (3)	0.0880 (8)	0.0247 (9)	
H3	0.4458	1.1096	−0.0481	0.030*	
C4	0.4227 (2)	0.9630 (3)	0.1440 (7)	0.0242 (9)	
H4	0.4602	0.9235	0.0441	0.029*	
C5	0.3768 (2)	0.9127 (3)	0.3447 (8)	0.0237 (8)	
H5	0.3836	0.8382	0.3852	0.028*	
C6	0.3200 (2)	0.9685 (3)	0.4915 (7)	0.0197 (8)	
C7	0.2704 (2)	0.9143 (2)	0.6992 (8)	0.0206 (8)	
C8	0.2146 (2)	0.9797 (3)	0.8361 (7)	0.0216 (8)	
C9	0.2103 (2)	1.0867 (3)	0.7638 (8)	0.0224 (8)	
H9	0.1731	1.1290	0.8562	0.027*	
C10	0.1592 (2)	0.9320 (3)	1.0447 (8)	0.0241 (8)	
H10A	0.1803	0.8685	1.1481	0.029*	
H10B	0.1448	0.9856	1.1986	0.029*	
C11	0.0402 (2)	0.8545 (3)	1.0569 (10)	0.0352 (10)	
H11A	0.0247	0.9072	1.2113	0.042*	
H11B	0.0596	0.7898	1.1601	0.042*	
C12	−0.0242 (2)	0.8243 (3)	0.8626 (10)	0.0373 (11)	
H12A	−0.0630	0.7929	0.9877	0.056*	
H12B	−0.0085	0.7715	0.7120	0.056*	
H12C	−0.0431	0.8888	0.7616	0.056*	
H1	0.245 (2)	1.209 (3)	0.503 (10)	0.055 (13)*	

### Atomic displacement parameters (Å^2^)

**Table:**

	*U*^11^	*U*^22^	*U*^33^	*U*^12^	*U*^13^	*U*^23^
O1	0.0336 (16)	0.0153 (11)	0.0306 (13)	−0.0011 (11)	0.0012 (14)	0.0050 (12)
O2	0.0230 (15)	0.0297 (13)	0.0313 (14)	−0.0058 (11)	0.0033 (13)	0.0058 (12)
N1	0.0239 (18)	0.0165 (13)	0.0271 (16)	−0.0001 (13)	−0.0047 (15)	0.0019 (14)
C1	0.021 (2)	0.0180 (16)	0.0205 (17)	−0.0020 (16)	−0.0043 (15)	−0.0005 (15)
C2	0.025 (2)	0.0178 (15)	0.0246 (17)	−0.0028 (15)	−0.0051 (19)	0.0070 (17)
C3	0.024 (2)	0.0262 (18)	0.0240 (17)	−0.0048 (17)	−0.0032 (18)	0.0027 (17)
C4	0.021 (2)	0.0256 (19)	0.026 (2)	0.0002 (16)	−0.0016 (17)	−0.0012 (16)
C5	0.030 (2)	0.0197 (16)	0.0212 (17)	0.0015 (16)	−0.0048 (18)	0.0027 (17)
C6	0.024 (2)	0.0180 (16)	0.0174 (17)	−0.0051 (15)	−0.0062 (15)	0.0007 (15)
C7	0.020 (2)	0.0183 (15)	0.0230 (18)	−0.0038 (15)	−0.0057 (16)	0.0024 (17)
C8	0.025 (2)	0.0195 (16)	0.0206 (17)	−0.0024 (15)	−0.0062 (17)	0.0022 (16)
C9	0.024 (2)	0.0202 (16)	0.0225 (17)	0.0029 (15)	−0.0022 (18)	−0.0017 (17)
C10	0.027 (2)	0.0228 (17)	0.0222 (17)	0.0001 (16)	−0.0001 (18)	0.0001 (17)
C11	0.039 (3)	0.028 (2)	0.038 (2)	−0.0043 (19)	0.016 (2)	0.002 (2)
C12	0.025 (2)	0.033 (2)	0.054 (3)	−0.0017 (17)	0.013 (2)	0.006 (2)

### Geometric parameters (Å, º)

**Table:**

O1—C7	1.260 (4)	C5—H5	0.9500
O2—C11	1.425 (4)	C6—C7	1.456 (5)
O2—C10	1.439 (4)	C7—C8	1.434 (5)
N1—C9	1.352 (4)	C8—C9	1.369 (4)
N1—C1	1.373 (5)	C8—C10	1.494 (5)
N1—H1	0.98 (4)	C9—H9	0.9500
C1—C2	1.402 (5)	C10—H10A	0.9900
C1—C6	1.408 (4)	C10—H10B	0.9900
C2—C3	1.374 (5)	C11—C12	1.504 (6)
C2—H2	0.9500	C11—H11A	0.9900
C3—C4	1.410 (5)	C11—H11B	0.9900
C3—H3	0.9500	C12—H12A	0.9800
C4—C5	1.373 (5)	C12—H12B	0.9800
C4—H4	0.9500	C12—H12C	0.9800
C5—C6	1.404 (5)		
			
C11—O2—C10	111.4 (3)	C9—C8—C7	119.3 (3)
C9—N1—C1	121.1 (3)	C9—C8—C10	119.5 (3)
C9—N1—H1	119 (3)	C7—C8—C10	121.2 (3)
C1—N1—H1	120 (3)	N1—C9—C8	123.4 (3)
N1—C1—C2	119.9 (3)	N1—C9—H9	118.3
N1—C1—C6	119.1 (3)	C8—C9—H9	118.3
C2—C1—C6	120.9 (3)	O2—C10—C8	108.3 (3)
C3—C2—C1	119.4 (3)	O2—C10—H10A	110.0
C3—C2—H2	120.3	C8—C10—H10A	110.0
C1—C2—H2	120.3	O2—C10—H10B	110.0
C2—C3—C4	120.8 (3)	C8—C10—H10B	110.0
C2—C3—H3	119.6	H10A—C10—H10B	108.4
C4—C3—H3	119.6	O2—C11—C12	108.6 (3)
C5—C4—C3	119.2 (3)	O2—C11—H11A	110.0
C5—C4—H4	120.4	C12—C11—H11A	110.0
C3—C4—H4	120.4	O2—C11—H11B	110.0
C4—C5—C6	121.7 (3)	C12—C11—H11B	110.0
C4—C5—H5	119.1	H11A—C11—H11B	108.3
C6—C5—H5	119.1	C11—C12—H12A	109.5
C5—C6—C1	117.9 (3)	C11—C12—H12B	109.5
C5—C6—C7	121.6 (3)	H12A—C12—H12B	109.5
C1—C6—C7	120.6 (3)	C11—C12—H12C	109.5
O1—C7—C8	123.1 (3)	H12A—C12—H12C	109.5
O1—C7—C6	120.4 (3)	H12B—C12—H12C	109.5
C8—C7—C6	116.5 (3)		
			
C9—N1—C1—C2	180.0 (3)	C1—C6—C7—O1	179.2 (3)
C9—N1—C1—C6	1.2 (5)	C5—C6—C7—C8	179.9 (3)
N1—C1—C2—C3	−178.9 (3)	C1—C6—C7—C8	−0.6 (5)
C6—C1—C2—C3	−0.2 (5)	O1—C7—C8—C9	−179.0 (3)
C1—C2—C3—C4	0.5 (5)	C6—C7—C8—C9	0.8 (5)
C2—C3—C4—C5	−1.0 (5)	O1—C7—C8—C10	−1.9 (5)
C3—C4—C5—C6	1.3 (5)	C6—C7—C8—C10	177.9 (3)
C4—C5—C6—C1	−0.9 (5)	C1—N1—C9—C8	−1.0 (5)
C4—C5—C6—C7	178.6 (3)	C7—C8—C9—N1	−0.1 (5)
N1—C1—C6—C5	179.2 (3)	C10—C8—C9—N1	−177.2 (3)
C2—C1—C6—C5	0.4 (5)	C11—O2—C10—C8	−179.7 (3)
N1—C1—C6—C7	−0.4 (5)	C9—C8—C10—O2	85.7 (4)
C2—C1—C6—C7	−179.2 (3)	C7—C8—C10—O2	−91.5 (4)
C5—C6—C7—O1	−0.3 (5)	C10—O2—C11—C12	179.6 (3)

### Hydrogen-bond geometry (Å, º)

**Table:**

*D*—H···*A*	*D*—H	H···*A*	*D*···*A*	*D*—H···*A*
N1—H1···O1^i^	0.98 (4)	1.78 (4)	2.707 (3)	157 (4)

Symmetry code: (i) −*x*+1/2, *y*+1/2, *z*−1/2.
